# Emotional Prosody Processing in Epilepsy: Some Insights on Brain Reorganization

**DOI:** 10.3389/fnhum.2018.00092

**Published:** 2018-03-13

**Authors:** Lucy Alba-Ferrara, Silvia Kochen, Markus Hausmann

**Affiliations:** ^1^Facultad de Ciencias Biomedicas, Austral University, Buenos Aires, Argentina; ^2^Estudios en Neurociencias y Sistemas Complejos, Consejo Nacional de Investigaciones Científicas y Técnicas (CONICET), Florencio Varela, Argentina; ^3^Science Labs, Department of Psychology, Durham University, Durham, United Kingdom

**Keywords:** prosody, temporal lobectomy, temporal lobe epilepsy, ictal semiology, dissociations, laterality

## Abstract

Drug resistant epilepsy is one of the most complex, multifactorial and polygenic neurological syndrome. Besides its dynamicity and variability, it still provides us with a model to study brain-behavior relationship, giving cues on the anatomy and functional representation of brain function. Given that onset zone of focal epileptic seizures often affects different anatomical areas, cortical but limited to one hemisphere, this condition also let us study the functional differences of the left and right cerebral hemispheres. One lateralized function in the human brain is emotional prosody, and it can be a useful ictal sign offering hints on the location of the epileptogenic zone. Besides its importance for effective communication, prosody is not considered an eloquent domain, making resective surgery on its neural correlates feasible. We performed an Electronic databases search (Medline and PsychINFO) from inception to July 2017 for studies about prosody in epilepsy. The search terms included “epilepsy,” “seizure,” “emotional prosody,” and “vocal affect.” This review focus on emotional prosody processing in epilepsy as it can give hints regarding plastic functional changes following seizures (preoperatively), resection (post operatively), and also as an ictal sign enabling the assessment of dynamic brain networks. Moreover, it is argued that such reorganization can help to preserve the expression and reception of emotional prosody as a central skill to develop appropriate social interactions.

## Importance of prosody assessment in epilepsy

Epilepsy is one of the most prevalent neurological conditions, and its prevalence is in the range of 5–10/1,000 cases worldwide (Sander, 2003). Besides the fact that epilepsy is a common neurological disorder, epilepsy has also been considered a privileged cognition model to study brain-behavior relationship. One of the most studied examples of brain functions mapping is language, a highly lateralized domain. Lateralization refers to the fact that each cerebral hemisphere has functional specializations. For example, several aspects of language such as syntax, semantics, and phonetics are dominantly processed in the left brain for the majority of right handed population. Instead, the right side of the brain is usually specialized in prosodic and pragmatic processing (e.g., Alba-Ferrara et al., 2011, 2012), domains that, although they have not been investigated as extensively as semantic processing, have a great impact in every day social life. Importantly, even if syntax and semantics are considered features of the left hemisphere, lateralization is not categorical. Instead, there is a continuum in the degree of lateralization (Knecht et al., 2002). In other words, language lateralization does not imply the absence of language processing in the non-dominant hemisphere.

Although overshadowed by its linguistic counterpart, emotional prosody processing is pivotal for oral communication and social interaction. Prosody constitutes a paralinguistic element of language and plays a prominent role in the organization of human communication. As described by Ross, prosody is a suprasegmental feature of language that conveys information beyond that transmitted by word choice or word order alone. The acoustic features associated with prosody include pitch, intonation, melody, cadence, loudness, timbre, tempo, stress, accent, and timing of pauses (Ross, 2000). To understand a speaker's intentions listeners must carefully attend to and decipher vocal inflections while analyzing the linguistic content of speech interpreting the relational significance of prosody (Rigoulot and Pell, 2012). It is known that successful comprehension of irony and sarcasm depends on the correct analysis of prosodic features together with semantic content. For example, sarcasm, a subcategory of verbal irony that communicates the speaker's negative or critical attitude, is often characterized by the hearer's recognition of the gap between the semantic content of the utterance and the speaker's communicative intent. Often, sarcastic utterances are statements that are absurdly inadequate or blatantly false in the light of reality or normative expectations (Matsui et al., 2016). Inability to comprehend prosody can result in poor social competence, contributing to isolation, constricted affect and diminished social satisfaction worsening patient's quality of life. Deficits in prosody processing have been vastly studied in disorders such as autism (Paul et al., 2005; Wang et al., 2007), schizophrenia (Alba-Ferrara et al., 2013; Rossell et al., 2013) and Parkinson disease (Benke et al., 1998; Pell and Leonard, 2003), and it has recently become of interest in epilepsy.

## Prosody/semantic double dissociations in epilepsy

In neuropsychology, a fruitful strand of research aiming to demonstrate the modularity of the mind consists in finding dissociations. Dissociations occur when a cognitive domain which is often seen as link to some other domain, results impaired whist the other remains intact. Double dissociations, occur when damage to one area of the brain causes impairment in a particular X domain but not in other Y domain, while damage to other brain area causes impairment in domain Y but not in domain X. Brain damage patient studies are well-suited to demonstrate double dissociation.

Besides the fact that prosody is based in speech as an intrinsic carrier, and speech also conveys semantic content, prosody can be disentangled from semantic processing. Double dissociations between emotional prosody and semantic processing have been reported in patients with unilateral lesions (Heilman et al., 1975; Ross and Mesulam, 1979). Considering the impairment in the expression of emotional prosody (Ross and Mesulam, 1979), and in the comprehension of emotional prosody (Heilman et al., 1975) that patients with right hemisphere brain damage suffer from, it was proposed that emotional prosody relies on the integrity of the right hemisphere (Ross, 1981). Further neuropsychological investigations have determined that patients with specifically right posterior lateral temporal lobe lesions have deficits in the comprehension (rather than the production or repetition) of emotional prosody (Ross and Mesulam, 1979), with patients with analogous lesions on the left producing near normal performance (Blonder et al., 1991).

There have been case studies reporting dissociation of prosody/semantic abilities in epilepsy literature. A case study reported a dissociation between semantic and prosodic abilities in epilepsy (Doherty et al., 1999). A seven years old child with Landau-Kleffner syndrome (with severe aphasia and epilepsy) showed normal emotional and propositional prosody production and comprehension according to her age, although the child was aphasic, and unable to produce and understand speech. She had preserved comprehension of environmental non-verbal sounds and acquired reading normally. Importantly, although in this particular case the location of the epileptogenic focus was not reported, Landau-Kleffner syndrome normally affects the left hemisphere (Doherty et al., 1999). A more recent review on postictal language functions concluded that patients with non-dominant temporal lobes partial seizures in the right hemisphere who are able to read during the ictal discharge, show however flattened prosody (Privitera and Kim, 2010). The same paper claims that postictal speech fluency dysfunction occurs when seizures originate or propagate to the dominant temporal lobe in the left hemisphere. Finally, a paper on healthy controls using inhibitory repetitive transcranial magnetic stimulation (rTMS) showed that when applying rTMS on the right temporal lobe reaction times (RT) where slower on a prosody decoding task, compared to sham and left rTMS; however, when doing a judgments on the emotional meaning of sentences (semantic task), rTMS on both sides caused slower RT compared to sham condition (Alba-Ferrara et al., 2012). Such finding is in line with the left temporal lobe as mediating linguistic/semantic processing. To summarize, epilepsy data add evidence to conclude that both semantic and prosodic processing (comprehension as well as production) are dissociated, can be independently impaired and are underpinned by different brain networks.

## Epilepsy as a model to study brain-cognition relationship

Epilepsy can lead to brain reorganization, which refers to the recruitment of neural networks previously not (or less) engaged in a given task, to compensate for directly lesioned or disconnected areas (Powell et al., 2007; Holopainen, 2008; Rosenberger et al., 2009; Enatsu et al., 2013). A long-term consequence of epileptic crisis is seizure-induced reorganization of synaptic circuitry. Alterations in synaptic connections may have several consequences, including changes in cognition or behavior. Temporal lobe epilepsy is one neurological disorder in which atypical language lateralization has high incidence as a consequence of brain reorganization. Such plasticity of brain networks involved in language processing in perinatal and early childhood injury has been vastly described (Cao et al., 1994; Isaacs et al., 1996; Muter et al., 1997; Stiles et al., 2003; Vandermeeren et al., 2003). Also, there are some cases of reorganization into atypical language lateralization following a later injury and or later surgery resection, even at the age of puberty (Hertz-Pannier et al., 2002). Although brain plasticity is particularly pronounced during early development/ontogenesis, it occurs throughout lifetime, even in older adulthood after brain injury. Mechanisms of brain plasticity after resection surgery of language-involved areas can explain patient's functional recovery (Duffau et al., 2003). There is evidence from magnetoencephalography showing intra- and interhemispheric reorganization of language brain network postsurgically (Pataraia et al., 2005), and further fMRI evidence suggests that contralateral brain network recruitment is a compensatory mechanism which can occur after resection surgery in the language dominant hemisphere, although it might be less effective that reorganization on the ipsilateral (damaged) side (Bonelli et al., 2012). The literature in this matter is still scant, protocol guidelines advise not to resect typical language areas due to the devastating effect it has on patient's functioning, and as a result few articles reported case studies or relative small samples.

Knowledge of language domains brain representation in epilepsy is of pivotal importance for two reasons. First, because in case of resistant epilepsy, resection of an eloquent area is not advisable (Rosenow and Lüders, 2001). Secondly, although some language domains such as prosody are not considered critical, they can be a clinically useful ictal sign offering cues on the localization and lateralization of the epileptic focus, of crucial importance for surgical planning (Bonora et al., 2011). Moreover, contrary to motor cortex and semantic processing networks, brain areas involved in prosody are not usually considered eloquent (i.e., critical for functioning). Importantly, guidelines do not prevent from resecting non-eloquent brain areas such as those underlying prosody processing. Thus, there might be bigger samples to study prosody following postsurgical brain reorganization compared to semantic processing.

One of the most practiced surgical interventions for resistant temporal lobe epilepsy consists in the unilateral resection of the anterior temporal lobe, which can include medial structures (e.g., amygdala and hippocampus) and partly, the lateral temporal lobes (parts of dorsal neocortical temporal gyri). Most of the evidence for prosody processing in epilepsy presented in this review will come from antero-temporal lobectomy intervention, which could affect emotional prosody processing, and particularly its comprehension. Moreover, there is additional evidence from split-brain patients, as callosotomy used to be a surgical treatment of epilepsy. To summarize, assessing prosody in epilepsy allow us to reveal how the neural correlates of this domain can be altered if the epileptogenic zone overlaps or propagates towards a node of the domain network, and how such network may get reorganized to be able to perform the task. Additionally, by resecting the epileptogenic zone and observe impairment (or not) in task performance in the aftermath, it is possible to establish unidirectional causality between a brain region and a behavioral output.

## Brain representation of prosody in epilepsy: presurgical studies

Epilepsy, particularly when the epileptogenic zone comprises the temporal lobules, very often entailed difficulties in emotional processing of visual stimuli (Meletti et al., 2003; Reynders et al., 2005). Vocal affect has not been assessed at the extension of its visual counterpart, although vocal affect conveys a huge amount of information essential for effective communication. At the same time, assessing prosody in epilepsy can give information about its brain representation by different means: (i) studying the semiology of the seizures, (ii) intracranial recordings (brain stimulation) and (iii) assessing the patients interictally. Assessing the semiology of seizures (i.e., spontaneous ictal activity or provoked by intracranial electrode stimulation) can provide information about physiological dysfunction in the epileptic network allowing to study a perturbed temporary state. Instead, studying the dysfunction caused by structural changes in the epileptic brain can offer information regarding permanent traits. Lastly, since assessing the semiology of seizures allows to study prosody production, evaluating the patients out of seizure allows studying not only the production but also the perception of prosody.

### Prosody production

There has been a couple of case studies reporting prosody production abnormalities during seizures in the non-dominant hemisphere (Montavont et al., 2005; Cercy and Kuluva, 2009). In the last case, video EEG recording showed a seizure onset in temporal areas of the right hemisphere, and disruption of emotional prosody production when the discharge spread towards the precentral and postcentral operculum (i.e., monotonous, hypophonic, emotionally neutral speech contrasting with the highly affective semantic content of the spoken utterances) (Montavont et al., 2005). Another report found an unusual high-pitched speech during ictal and interictal discharges in right frontal and fronto-temporal areas. In addition, the cited case of gelastic epilepsy also presented ictal laughter (Cercy and Kuluva, 2009). Although the neural representation of laughter overlaps humor and emotion processing, it is non-conclusive whether this kind of stereotyped ictal laughter carries any affect. Laughter with mirth, which comprises not only a stereotyped motor response but also an emotional outburst, has been described as a consequence of electrical stimulation of the pregenual anterior cingulate cortex (pACC) in a group of patients with epilepsy (Caruana et al., 2015). Although the lateralization of laugher with mirth was unclear (i.e., left for some of the patients and right for others), only direct stimulation of the pACC provoked the response. Ictal activity and brain stimulation allow us to have a direct measurement and to establish a causal relation between brain and behavior, but sample sizes of this kind of studies are relatively small. Instead, assessing the behavior in patients with structural brain abnormalities gives the opportunity to access to bigger samples.

An elegant avenue of testing a causal relation between brain representation, and behavior and cognition such as language, is given by the Wada test (Wada and Rasmussen, 1960). In a historical paper, Ross and colleagues (Ross et al., 1988) manipulated speech and prosody production by anesthetizing the left and right hemisphere alternatively in a group of epileptic patients with an intracarotid injection of sodium amobarbital as a presurgical evaluation of hemispheric functioning for potential resection. Speech and prosody production were evaluated at baseline (before drug injection) and then again during anesthesia of the left and right cerebral hemispheres while patients are awake and responsive. All patients in this study manifested a left non-fluent aphasia during injection of sodium amobarbital in the left carotid. On the contrary, during anesthesia of the right hemisphere, all patients showed severe affective flattening of spontaneous speech. Such findings strongly supported the idea of a double dissociation of speech and emotional prosody in the left and right hemispheres, respectively.

### Prosody recognition

Epilepsy is very often associated with damage to brain structures within the epileptogenic zone (EZ). Fowler et al. (2006) investigated prosody recognition in adults with temporal lobe epilepsy and unilateral amygdala damage, by comparing patients with left and right damage. The authors found that although none of the groups differed from controls in prosody comprehension, some patient's individual performance were impaired (Fowler et al., 2006). Specifically, moderate to severe impairment (z score < 2.33) in sad and happy prosody was found in 2 out of 3 right amygdala damage patients since none of the left amygdala damage patients showed impaired performance. On the other side, moderate impairment in anger prosody was found in 2 out of 4 left amygdala damage patients since none of the right amygdala damage patients showed impaired performance. The task employed consisted in labeling spoken digits with different emotional intonations. Within these individual cases, two individuals were right and other two left damaged. It is important to note that epilepsy onset of the tested patients occurred during infancy which might have given place to brain reorganization. A recent study assessing children with temporal lobe epilepsy at emotional prosody recognition (half of them with left epileptogenic zone, with or without MRI structural lesion) did not show prosody task performance differences between the right and left epilepsy groups (Laurent et al., 2014). Importantly, the cited study applied a task in which participants had to recognize a target emotion from a choice of two voices (using pseudo-words sentences spoken with either happy and surprised prosody or angry and sad prosody). Also investigating temporal lobe epilepsy and emotional prosody comprehension, another study in adult patients with variable age of onset and positive MRI structural findings (most of them with mesiotemporal sclerosis) found deficits in both patient groups (left and right epilepsy) compared to healthy controls, although left and right groups did not differ from each other (Kho et al., 2008). Noteworthy, Kho et al. applied semantically neutral sentences spoken in angry, sad, happy and disgust prosody. A recent report showed that patients with mesiotemporal lobe epilepsy (both hemispheres compromised) had impaired performance in emotional prosody recognition compared to healthy controls and also to a case of Urbach-Wiethe disease with bilateral amygdala atrophy (Meletti et al., 2014). Impaired performance for anger and fear vocal emotions was already described in an epilepsy case with bilateral amygdala damage (Scott et al., 1997). The patient, whose epilepsy started during adulthood, understood the concept of emotion (i.e., patient described circumstances which triggered emotions), but was unable to identified emotions conveyed by the tone of voice in semantically neutral sentences. Finally, it should be noted that patient's emotional status has an impact on prosody comprehension. Epilepsy very often co-occurred with mood disorders (Kanner, 2007), and it has been shown that depressed epilepsy patients performed worse than euthymic epileptics in prosody recognition, although not in facial emotion recognition (Brand et al., 2009). Further research should address specific effects of co-morbid mood disorders on particular valences and arousal.

In summary, it can be conclude that in epilepsy (a) age of onset is determinant for prosodic abilities, as an early onset (during infancy) may facilitate brain plasticity and the subsequent recruitment of alternative neural networks to cope with the task. (b) According to studies assessing epilepsy out of seizures, patients with bilateral amygdala damage are particularly compromised compared to unilateral damage. (c) the nature of stimuli, that is whether it also contain semantic meaning in addition to prosodic intonation, might determine the involvement of additionally involved brain regions. Finally, it should be noted that the vast majority of neuroimaging and neurostimulation studies in healthy subjects which investigated emotional prosody (production and perception) localized emotional prosody dominantly in the right hemisphere (e.g., Bryden and MacRae, 1988; Kotz et al., 2003; Friederici and Alter, 2004; Alba-Ferrara et al., 2012). Additionally, studies on the semiology of seizures and intracranial recordings and stimulation, pointed out that the right hemisphere is critical for emotional prosody processing. One possibility is that while the right temporal lobe is affected, recruitment of the contralateral left hemisphere for prosody recognition might suffice to compensate, resulting in an adequate performance for the task. If both temporal lobes are compromised, compensatory neural recruitment might be prevented. Following this line of thoughts, patients with unilateral left temporal damage should be able to process prosody normally. However, Fowler et al. (2006), reported two patients with unilateral left temporal damage impaired at prosody processing. It should be noted that one of those patients had abnormal EEG activity bilaterally, which may imply that although the structural lesion is unilateral, the “functional” lesion spreads towards the right. The other patient showed an overall emotion processing impairment, as revealed by poor performances at detecting facial emotion, emotional meaning in sentences as well as emotional prosody, as well as a relatively low premorbid IQ, which might explain his general poor performance in all tasks, including prosody perception. Finally, as pointed out by Alba-Ferrara and colleagues, the left temporal lobe might not be crucial for the basic task of emotional prosody decoding, but when the stimuli applied also conveys semantic content such as in sentences, prosodic and semantic processing might be more difficult to disentangle and both temporal lobes may get involved (Alba-Ferrara et al., 2012). Stimuli free from semantic content may be a more appropriate to assess the laterality of emotional prosody processing.

## Brain representation of prosody in epilepsy: post-surgical studies

There are some reports on postsurgical prosody processing showing specific impairments in subgroups of patients (Brierley et al., 2004; Dellacherie et al., 2011; Prete et al., 2014; Milesi et al., 2015). For example, after unilateral anterior temporal resection including the amygdala, Milesi and colleagues showed a general emotional prosody deficit in patients, regardless of the side of surgery. Similar results were found on a study of unilateral amygdala lesion for fear and surprise valences, in which left and right side lesion groups did not differ between themselves, but performed significantly worse than matched controls (Dellacherie et al., 2011). Interestingly, in the study by Milesi and colleagues, patients with left side surgery (four in total) rated the emotional stimuli as more intense than the only right side surgery patient included, who presented a specific deficit at recognizing fearful prosody, but other valences were not affected (Milesi et al., 2015). Importantly, the patient's lesion was located in the right anterior temporal lobe including the amygdala, but had preserved planum polare, STG and STS. A similar study also found that fearful voices might be the most compromised emotional prosody after anterior temporal lobectomy in patients with unilateral (left and right) as well as bilateral amygdala damage (Brierley et al., 2004). To interpret postsurgical data and the effect of some region of interest resection, it might be necessary to look into clinical cases in which a brain structure is damaged. Two independent reports on single cases of Urbach-Wiethe disease (which causes bilateral amygdalae damage and epilepsy) demonstrated that patients were unimpaired in prosody recognition, although they were unable to recognize facial emotion (Adolphs and Tranel, 1999; Meletti et al., 2014). Meletti and colleagues compared the Urbach-Wiethe disease patient to healthy controls and patients with bilateral TLE, finding this last group impaired at emotional prosody recognition compared to controls and to the single case patient. The authors proposed that prosody recognition depends on extra-amygdalar temporal structures, especially in the right hemisphere, as solely amygdala damage does not suffice to cause deficits. Regarding frontal regions, a single case study reported normal speech prosody production after ablation of the right orbitofrontal cortex and frontal pole, although amusia was found implicating separated neural mechanisms for both domains (McChesney-Atkins et al., 2003). The temporal cortex, including the STS, STG, temporal pole and also amygdala, particularly in the right hemisphere, appear to be pivotal nodes underlying prosody processing, the involvement of the frontal lobes is less clear.

A few studies compared emotional prosody performance in patients before and after surgery, shedding light into the impact that ablation of prosody nodes has on performance (Kho et al., 2008; Berberian et al., 2015). One of the studies assessed patients with unilateral temporal lobectomy, mostly in the left hemisphere, and did not find changes between pre-surgical and post-surgical performances on spontaneous prosody production (Berberian et al., 2015). Noteworthy, this study is the only one comparing pre and post-surgical performance in prosody production instead of prosody recognition. Unfortunately, the cited study did not include a healthy control group to test whether patient's performance was normal. Another study compared patients with left side and right side unilateral anterior temporal lobectomy and found no changes in emotional prosody recognition performance after surgery, neither group differences before or after surgery, although both patients group performed less well in emotional prosody than healthy controls before and after surgery (Kho et al., 2008). Performance was not affected by surgery neither there were differences between the left and right group. Although the authors interpreted that perhaps the resected nodes where nor essential for the prosody task, alternatively, it could be inferred that those patients, having the epileptogenic zone coincident with putative nodes for prosody processing, might have recruited a non-putative but healthy brain network to perform the task even before surgery, suggesting that epilepsy itself results in brain plasticity. Following this line of thought, it has been shown that in left TLE the right temporal lobe activated for reading comprehension test after surgery (left anterior temporal resection) (Noppeney et al., 2005). It is likely that plastic changes following epilepsy results in a more bilateral representation of typically lateralized functions.

So far we have discussed several studies evaluating emotional prosody processing in epilepsy which outcomes are multifarious. Due to the complexity of the topic, and considering epilepsy as a syndrome clinically and etiologically heterogeneous, a comparative table was elaborated. To elaborate this table, a Medline search (Pubmed) was conducted, and work published with “epilepsy” and “prosody” or “vocal affect” terms. Table 1 summarizes a list of studies on emotional prosody in epileptic patients; describing the lesions, the type of epilepsy, the emotional prosody paradigms applied for assessment, and the outline of the main results.

**Table 1:** List of studies fulfilling the inclusion criteria of the review.

**Studies**	**MRI lesion location (sample size)**	**Surgery type**	**Type of epilepsy (EZ)**	**Type of prosody task**	**Performance**	**Time of assessment**
Adolphs and Tranel, 1999	Bilateral amygdalae damage (2), right amygdala damage (2), left amygdala damage (5)	All unilateral lesions had temporal lobectomy with partial or total amygdala resection	Bilateral patients: Urbach-Wiethe, Herpes simplex. Unilateral patients: TLE	Emotion labeling of sentences spoken in affective valences	Unimpaired performance of all groups	Post-surgical
Berberian et al., 2015	Pre-surgical group: Left (14), right (9), Bilateral (8). Postsurgical group: Left (14), right (9), bilateral (8)	Temporal lobectomy	TLE	Speech evaluation by a speech therapist (prosody production)	No difference between pre and post-surgical performance	Pre and post-surgical (cross-sectional)
Brand et al., 2009	Heterogeneous group (32)	Heterogeneous	Heterogeneous	Comprehensive Affect Testing System (CATS-A)	Epilepsy patients in comorbidity with depression performed worse that epilepsy patients without depression	Pre-surgical
Brierley et al., 2004	Antero-temporal resection (13 left, 15 right)	Unilateral anterior temporal lobectomy	TLE	Emotion labeling of utterances spoken in affective valences	All patients have difficulties with fear prosody. Patients with bilateral amygdala damage had poorer performance overall.	Post-surgical
Cercy and Kuluva, 2009	Mild periventricular hyperintensity (1)	None	Gelastic epilepsy. Right frontal ZE	Clinical evaluation of ictal speech	Ictal dysprosodia	Pre-surgical
Dellacherie et al., 2011	Left TLE (5 out of 10 complete resection of amygdala). Right TLE (3 out of 4 complete resection of amygdala)	Unilateral anteromedial temporal lobe resection (10 left, 10 right)	TLE	Judgment of intensity, valence and arousal of vocalizations (Montreal Affective battery)	Impaired fear and surprise prosody recognition in both patients groups compared to controls. No difference between patient Groups	Post-surgical
Fowler et al., 2006	Asymmetrical amygdala damage (13 right, 15 left)	Pre-surgical study	Right and left TLE	Emotion labeling of verbal and nonverbal prosodic sounds	Inconclusive. No group differences. Some patients have prosody difficulties	Pre-surgical
Frühholz et al., 2015	Mesio-temporal lesions (10 right, 10 left)	Unilateral temporal lobectomy	MTLE	Judgment of binaural prosodic vocalizations with attention directed toward or away from prosody on one side	Behaviorally unimpaired. Only right MTLE recruited contralateral amygdala and auditory cortex for prosody processing (fMRI study)	Post-surgical
Kho et al., 2008	Majority of patients with mesio-temporal sclerosis (32 total)	Anterior temporal resection	Unilateral TLE (half left)	Emotion labeling of sentences spoken in affective valences	Presurgically, both patient groups performed worse than control at prosody recognition. Unchanged after surgery	Pre and post-surgical evaluation
Laurent et al., 2014	21 out of 39 patients with mesial abnormalities	Pre-surgical study	Unilateral TLE (half left)	Emotion labeling of sentences spoken in affective valences	Unimpaired emotional prosody. Left and right group did not differ from each other	Pre-surgical
McChesney-Atkins et al., 2003	Right subfrontal craniopharyngioma (1)	Right subfrontal, orbitofrontal and frontal pole resection	Right frontal	Clinical evaluation of ictal speech	Normal prosody processing, although some pitch processing impairment	Post surgical
Meletti et al., 2014	Bilateral amygdalae damage (1)	None	Urbach-Wiethe/MTLE	Emotion labeling of sentences spoken in affective valences	Unimpaired	n/a
Milesi et al., 2015	Anterior temporal (4 left, 1 right)	Anterior temporal resection	TLE	Emotion labeling of prosodic non-verbal sounds	General impairment of emotional prosody. Left TLE rated emotion as more intense than right TLE. Right TLE had a specific fear prosody deficit	Post-surgical
Montavont et al., 2005	None (1)	Intracranial recordings	Right MTLE	Clinical evaluation of ictal speech	Ictal dysprosody and altered prosody during right frontal operculum stimulation	Intracranial recording.
Prete et al., 2014	Total absence of callosum (1), partial absence of callosum (1)	Complete callosotomy, partial callosotomy	Not specified	Judgment of emotional intonation of syllables in a dichotic listening task	Total callosotomy patient scored higher on happy prosody presented to the right than to the left ear. He scored lower to sad prosody presented to the left than the right ear	Post-surgical
Ross et al., 1988	Not specified. Wada test study (5)	Pre-surgical study	Not specified	Clinical evaluation of speech during Wada test	Inability to produce emotional prosody after right Wada test. Aphasia after left Wada test	Pre-surgical
Scott et al., 1997	Bilateral amygdalae damage (1).	None	TLE		Impaired at fear and anger prosody perception	Pre-surgical
Turner et al., 2015	Not specified (10)	None	Epilepsy-aphasia syndrome (GRIN2A mutation)	Clinical evaluation of speech.	Impaired pitch and prosody	n/a

*EZ, epileptogenic zone; TLE, temporal lobe epilepsy; MTLE, medial temporal lobe epilepsy*.

Finally, an fMRI study on emotional prosody comparing patients with left and right amygdala resection (without including a group of healthy controls) revealed that, although left and right side epilepsy groups performed similarly on the behavioral level, they engaged the auditory cortex differently (Frühholz et al., 2015). In a typical dichotic listening task, two different stimuli are presented simultaneously (one on each ear) and the participant should inform the heard stimuli (non-forced condition). Left ear advantage (LEA) reflected by stimuli on the left side being preferentially reported, is expected for prosodic processing, and a right ear advantage (REA) is expected for semantic processing (Bryden and MacRae, 1988). In a dichotic emotional prosody task designed by Frühholz and colleagues, participants had to focus on one ear ignoring the opposite and classify the heard stimulus. The authors analyzed all trials together (attending either the right or left ear) and found that patients with right amygdala resection had enhanced bilateral auditory cortex and left amygdala activation in response to prosody, whereas left amygdala resection patients did not show such contralateral effect (Frühholz et al., 2015). Although the authors interpret that amygdala damage leads to decreased ipsilateral activation, and only left amygdala damage impaired the cortical response to emotional prosody showing the critical role of the left amygdala in the task, two alternative explanations are possible. Firstly, it should be noted that performance was similar in both groups, perhaps reflecting that unilateral medial lobectomy including the amygdala did not include the necessary structures to cause a deficit, which might be located more lateral and posterior to the resected areas, such as the STS, and STG. Secondly, the results might reflect a more efficient processing of emotional prosody in left damage patients, as they can perform the task without recruiting contralateral areas, which would reflect a prominent role of right temporal areas for emotional prosody processing. In other words, having a preserved right amygdala and right anterior temporal cortex may be sufficient to process prosody, whereas impairment in such pivotal nodes leads to extra recruitment of the contralateral side to compensate. The study by Frühholz et al. (2015) might also suggest a pivotal role of interhemispheric connectivity in the laterality of emotional prosody, as it showed that ablation of one node may result in recruitment of homologous areas on the contralateral side probably via callosal fibers.

Importantly, the present work does not claim that emotional prosody processing is the only cognitive domain compromised in epilepsy. The neurocognitive profile of epilepsy patients differs largely depending on the cause of the syndrome, topography of epileptogenic areas, pathogenetic mechanisms involved, and the diverse features characterizing its clinical course. Localization-related cryptogenic and symptomatic epilepsy disorders are accompanied by focal deficits that mirror the specific functions of the respective areas (Elger et al., 2004). It should be noted that higher cognitive function such as attention, working memory, processing speed and related executive functions may have an impact in prosodic tasks performance; and particularly patients with right hemisphere damage might have difficulties to filter out distractor in case in valence incongruity on top of prosody processing deficits (Bowers et al., 1987). However, the present review selected studies in which general cognition was not severely affected or not compromised to the same extent than prosody in order to make inferences about the neural correlates, organization and reorganization of emotional prosody processing.

## The role of interhemispheric crosstalk in emotional prosody

The corpus callosum is the most extended fiber bundle in the brain, connecting mainly homotopic areas of the left and right cortices (Hausmann and Güntürkün, 2000). In a case study of a patient who had a lesion in the anterior part of the corpus callosum, while the cortices were not affected, an aprosodia (i.e., inability to produce tonal variation in speech) was found (Klouda et al., 1988). An interesting case to study interhemispheric crosstalk is callosal agenesis. To understand its implications, it should be noted that during cerebral development the brain undergoes reorganization, allowing for compensatory mechanisms, meaning that callosal agenesis patients have also been shown to be capable of some sort of intra and interhemispheric transfer (although much slower) (van der Knaap and van der Ham, 2011). A group of adults with callosal agenesis was found to be unable to comprehend proverb meaning and emotional prosody compared to matched controls while people with callosal agenesis had no problems in understanding literal language (Paul et al., 2003). The authors suggested a direct link between the functional difficulties, including emotional prosody deficits, and agensis of the corpus callosum, and consequently lack of interhemispheric integration. Noteworthy, the group of people with callosal agenesis revealed language impairments similar to those of patients following right hemisphere damages (Paul et al., 2003). Non-literal language and emotional prosody processing seem to require efficient interhemispheric interaction, as each half makes a unique contribution to paralinguistic interpretation. Since the right hemisphere is needed for processing prosodic information, the left hemisphere may be needed to make a judgment and classify emotional valence.

Further evidence comes from patients with callosotomy—a surgical resection of the corpus callosum to stop seizure propagation. A recent report showed that a patient with partial callosotomy (only the anterior part of the callosum was cut) performed similarly than controls in a dichotic listening task, using syllables spoken with sad and happy prosody. On the contrary, a patient with complete callosotomy showed a left hemispheric superiority for emotional prosody processing in the same task (Prete et al., 2014). It is possible that partial callosotomy of the anterior portion does not impact on the performance on the task, as prosody comprehension nodes are more located in posterior areas, such as the temporo-parietal junction and the posterior part of the STG (Alba-Ferrara et al., 2011, 2012). Reduced laterality in prosody processing due to complete callosotomy is expected, as the right hemisphere (superior for prosody) may be unable to transfer information to the left hemisphere in order to produce a response (labeling, judgment, etc.). However, the complete callosotomy case presented by Prete and colleagues showed reversed laterality. Unfortunately, as this study was performed only postsurgically, it is not possible to know whether the patient was typically lateralized before callosotomy. Alternative explanations about the effects of callosotomy on prosody processing should also be considered, as it is difficult to infer whether callosal fibers predominantly have an inhibitory or excitatory role in the transfer of linguistic computations in the brain. If such connection are inhibitory, callosotomy could result in a redundant dual processing interfering with a more efficient unilateral processing (Hirnstein et al., 2008).

## Summary

Emotional prosody, a lateralized domain, can be affected in epilepsy, resulting in poor social cognition and diminishing quality of life. Emotional prosody brain representation mirrors speech processing on the contralateral side. Although epilepsy is a complex syndrome impacting on several brain networks beyond the epileptogenic zone (EZ), it predominantly affects one specific hemisphere. The treatment for drug-resistant epilepsy consists in the resection of the EZ. Abnormal ictal prosody production can be a sign offering hints on the localization of the epileptogenic network. In other words, aberrant prosody production within seizures indicates an overlap between the neural correlates of both prosody production and epilepsy. For prosody production, video EEG shows prosodic disruption during right frontal and temporo-frontal areas. This laterality finding was also confirmed by Wada test. Regarding emotional prosody comprehension, although there are some factors impacting laterality such as contralateral compensatory mechanisms, characteristic of the stimuli (whether it is pure prosody or it conveys semantic content) and age of onset (as it influences plasticity), it is plausible that the right posterior temporal lobe has a prominent role in such function. Evidence from postsurgical studies should be taken cautiously, as it is possible that, when the putative prosody processing network is affected by epilepsy, brain plasticity occurs. If epilepsy itself drives brain reorganization of prosody processing (before surgical intervention), it would be expected that that right TLE patients might not be severely affected in this function, as they might compensate with recruitment of alternative brain networks. Then, surgery on the putative prosody network would not have such a clear effect on postsurgical prosody performance, even compared to presurgical performance. Although there is scant evidence comparing prosody processing pre and postsurgical performances in epilepsy, most of postsurgical studies show poorer performance in patients with right temporal lobectomy or in both groups of patients (right and left lobectomy) compared to healthy controls. None of the studies found poorer performance in the left lobectomy group compared to right lobectomy. Thus, it is possible that besides the existence of seizure-driven reorganization of prosody comprehension in right TLE, such reorganization cannot be thought in absolute terms, and putative brain representation partly remains in TLE. Finally, data on intherhemispheric crosstalk suggests that both hemispheres have differential roles in prosody processing, and since the right hemisphere is preponderant for the task per se, left hemisphere contributions are needed for categorizing and labeling emotions.

## Further research directions

So far, this review has addressed certain brain nodes without taking into account their connectivity. However, ictal activity rapidly propagates, reflecting recruitment of connected areas which makes it difficult to establish a causal relation between a single brain node and its behavioral correlate. On the other hand, seizure propagation offers an opportunity to study brain networks and how different nodes interact to carry out certain task. New functional connectivity techniques can identify areas with the highest connectivity to the epileptogenic focus, offering a picture of seizure propagation, even when measured interictally (Boerwinkle et al., 2016). Up to now, however, there are no studies assessing the functional connectivity of emotional processing in epilepsy yet. We foresee further research blossoming in this area.

## Author contributions

LA-F: wrote the article; MH and SK: revised and supervised the manuscript; SK and MH: contributed equally to the article and share senior authorship.

### Conflict of interest statement

The authors declare that the research was conducted in the absence of any commercial or financial relationships that could be construed as a potential conflict of interest.
